# Association Between Caffeine Levels and Symptom Profile in Schizophrenia: Results from a Cohort Study in Central Greece

**DOI:** 10.3390/brainsci16020209

**Published:** 2026-02-10

**Authors:** Michel B. Janho, Maria N. Papaliaga, Athina A. Samara, Stamatia Papoutsopoulou, Matthaios Speletas, Nikolaos Christodoulou, Eftihia Asprodini

**Affiliations:** 1 Department of Clinical Pharmacology, Faculty of Medicine, University of Thessaly, 411 10 Larisa, Greece; 2 Department of Psychiatry, University Hospital of Larissa, 411 10 Larisa, Greece; 3 Faculty of Public and One Health, University of Thessaly, 431 00 Karditsa, Greece; at.samara93@gmail.com; 4 Department of Biochemistry and Biotechnology, School of Health Sciences, University of Thessaly, 413 34 Larissa, Greece; stapapou@uth.gr; 5 Department of Immunology and Histocompatibility, Faculty of Medicine, University of Thessaly, 382 21 Volos, Greece

**Keywords:** caffeine, schizophrenia, PANSS, cognition, ACE-R, symptoms

## Abstract

**Highlights:**

**What are the main findings?**

- Elevated caffeine serum levels are correlated with impaired attention.
- Violent behavior is seen more commonly as caffeine serum levels increase.

**What are the implications of the main findings?**

- Caffeine may have a paradoxical effect on attention in patients with schizophrenia.
- The introduction of routine caffeine monitoring may help manage patients prone to aggressive behavior.

**Abstract:**

Introduction: Caffeine is the most consumed psychostimulant worldwide. Schizophrenia is an uncommon mental disorder affecting 0.34% of the global population. The aim of the current study was to investigate a possible association between caffeine consumption and symptom profile, dangerous behavior, and cognitive functions in patients with schizophrenia. Methods: This prospective cohort study included consecutive patients diagnosed with schizophrenia who were admitted to the psychiatry ward or visited the psychiatry outpatient clinics at a tertiary University Hospital in Greece for a period of 12 months. All patients underwent an extensive psychiatric and cognitive function assessment using the standardized Greek version of the Positive and Negative Symptom Scale (PANSS), the generalized anxiety disorder scale (GAD-7), and the Addenbrooke cognitive test (ACE-R). Results: In total, 53 patients were included in the present study. Mean age of the participants was 45 ± 11 years. The mean age at onset was 23 ± 7 years, while mean duration of illness from age of onset was 21.58 years. Caffeine serum levels exhibited a positive correlation with the poor attention component of the general psychopathology PANSS subscale, as well as with the attention and orientation component in the ACE-R. Moreover, another positive correlation was observed between the perilous behavior PANSS subscale and caffeine serum levels. Conversely as caffeine serum levels increased, fewer negative symptoms were reported, specifically, the poor rapport and passive/apathetic social withdrawal of the negative PANSS subscale. Conclusions: In summary, this study highlights the significant associations between caffeine serum levels, symptom severity, and cognition among patients with schizophrenia. While the findings provide valuable insights, they should be interpreted with caution due to the study’s several limitations. More larger scale cohort studies are needed in order to elucidate the impact of caffeine consumption in patients with schizophrenia.

## 1. Introduction

### Background/Rationale

Caffeine is the most consumed psychostimulant worldwide [1]. It is naturally found in coffee, tea, and cocoa, while it can also be found in chocolate, energy drinks, and caffeinated soft drinks [2,3]. Coffee and tea are the most consumed caffeine containing beverage amongst adults while chocolate, energy drinks and caffeinated soft drinks are consumed mainly by adolescents and children [4,5]. Recent worldwide statistics showed that global coffee consumption amounted to approximately 177 million 60 kg bags of coffee in 2023/2024, a 2.3% increase from the previous year [6].

Consuming caffeine results in 99% absorption after about 45 min [7,8,9]. Delayed absorption of caffeine, however, is observed after cola or chocolate consumption [10]. Caffeine can pass through all biological membranes owing to its hydrophobic properties [11] and, therefore, can pass freely through the blood–brain barrier [12]. Moreover, the pharmacokinetics of caffeine in blood and cerebrospinal fluid are similar [13]. Caffeine is metabolized in the liver mainly (95%) by CYP1A2 [14] into its main active metabolite paraxanthine [15] and to a lesser effect by CYP3A4, xanthine oxidase, and N-acetyltransferase 2 [16,17]. Other known metabolites are theobromine and theophylline, which are also biologically active. A plethora of factors can influence CYP1A2 activity and ultimately contribute to variations in caffeine metabolism. These include gender, race, single nucleotide polymorphisms, and exposure to inhibitors/inducers (e.g., smoking) [1,14,15,18]. The metabolites are excreted in urine [15] alongside less than 2% of unchanged caffeine [19].

Schizophrenia is an uncommon mental disorder affecting 0.34% of the global population, according to the latest 2023 IHME global burden of disease study [20]. Although often thought to affect men and women equally, schizophrenia occurs marginally more frequently in men than in women (male-to-female incidence rate ratio of 1.7 [95% CI 1.46–1.97]) [21]. The main clinical features of schizophrenia include positive symptoms or reality distortion symptoms (e.g., delusions, hallucinations, and thoughts of suspiciousness), negative symptoms (e.g., lack of volition, reduced speech output, and blunt affect), and the disorganization syndrome (e.g., disorganized behavior and the uncommon symptom of inappropriate affect) [22,23]. Cognitive impairment is now considered an additional clinical feature of schizophrenia [24]. This impairment varies greatly in degree, as reported by a plethora of studies, ranging from little [25] to none [26] to large deficits across memory, orientation, and general knowledge domains [27]. Moreover, distinct limitations are observed in executive function, memory, and sustained attention [28,29]. The pathogenesis of schizophrenia has been hypothesized as a dysfunction of the dopamine [30,31] or glutamate [32,33] systems in the brain. Recently, the purinergic hypothesis of schizophrenia has emerged, proposing that reduced adenosinergic activity may underlie and integrate previously suggested mechanisms of the disorder [34].

The stimulant effects of caffeine on the central nervous system can be explained by its competitive antagonism against endogenous adenosine on A1 and A2A adenosine receptors [35,36,37]. Stimulation of the central nervous system at low caffeine doses (100 mg) increases locomotor activity [1,38] as well as arousal [39] but can manifest anxiety at higher doses (500 mg) [40,41]. Furthermore, improvements in cognitive functions have been reported after caffeine consumption on a regular basis, namely reaction time [42,43] and verbal memory [42,44]. On the other hand, acute administration of caffeine has been reported to improve sustained attention [45,46].

Adenosine, on the other hand, functions as a neuromodulator in both the dopaminergic and glutamatergic systems [47]. Specifically, adenosine A1 and A2A receptors form heteromers with dopamine D1 and D2 receptors in the striatum [48,49], resulting in the inhibition of dopamine signaling [50] as well as decreasing dopamine D2 affinity for agonists [51]. Adenosine modulates glutamate transmission in a concentration-dependent manner in the corticostriatum; low adenosine concentration inhibits glutamate release via the adenosine A1 receptor, and high adenosine concentration leads to glutamate potentiation via activation of the adenosine A2A receptor, which in turn inhibits the A1 receptor and its aforementioned inhibitory effects on glutamate release [52,53]. The hypothesized hypofunctioning of the adenosinergic system in schizophrenia implies that the interaction of adenosine/dopamine heteromers as well as adenosine/glutamate heteromers via glutamate metabotropic receptors (mGluRs) [54,55] are hindered to a larger extent by the antagonistic effect of caffeine on adenosine receptors, thus enhancing this hypofunctioning and worsening schizophrenia symptoms. Consequently, many attempts have been made to reverse engineer medications based on this interaction to treat schizophrenia but without solid evidence [56].

Caffeine consumption by patients with schizophrenia can reach doses as high as 500 mg/day based on real-world data reports [57,58]. Conversely, daily doses up to 400 mg/day (around 2–4 cups of coffee or up to 6 cups of tea) are considered safe in non-pregnant adults according to the scientific opinion by the European Food Safety Authority (EFSA) [14]. Beyond this limit, caffeinism can occur which can manifest as nausea, vomiting, tachycardia, tachypnea, depressed consciousness, irritability, and headache. The implications, following profound caffeine consumption in this subpopulation, are well documented and they include cases of de novo psychosis [59,60] or deterioration of stable patients with schizophrenia [61,62]. Moreover, data regarding the effects of caffeine on cognition in patients with schizophrenia are sparse with mixed results [63,64].

Serum caffeine levels in patients with schizophrenia have not been extensively investigated in the current literature and, except for a few solitary case reports where lethal caffeine levels ranged from 80 mg/L to 100 mg/L [65,66,67,68], there are no standardized caffeine serum levels generally accepted as normal. A double-blind placebo-controlled study of patients with schizophrenia reported increased scores on psychopathology scales such as thought disorder, unusual thought content, and euphoria activation after acute administration of caffeine (10 mg/kg) [69]. A 2004 case control study reported higher serum caffeine levels in patients with schizophrenia versus healthy controls, but this difference was present only among smokers [70]. A few studies, on the other hand, have examined the relationship between caffeine serum levels and illness severity in patients with schizophrenia [69,71], while others examined reported caffeine intake with illness severity [72,73] with conflicting results and recommended more precise measures of caffeine intake [73].

Thus, given the knowledge gap in the literature accompanied by the mixed outcomes, the aim of the current study was formed to investigate any possible associations between caffeine consumption and symptom profile, dangerous behavior, and cognitive functions in patients with schizophrenia.

## 2. Methods

### 2.1. Study Design

This was a prospective cohort study including patients diagnosed with schizophrenia according to ICD-10 diagnostic criteria. Subjects with schizophrenia who are followed up on a regular basis at the outpatient clinic were enrolled.

### 2.2. Setting

This study recruited all consecutive patients who were admitted to the psychiatry ward or visited the psychiatry outpatient clinics at a tertiary University Hospital in Greece for a period of 12 months (June 2023–May 2024).

### 2.3. Participants

Eligibility criteria included adult patients aged 18 to 70 years old suffering from all subtypes of schizophrenia. The exclusion criteria were a personal history of alcohol/substance use disorder, severe physical illness, or pregnancy.

### 2.4. Data Sources/Measurements

Demographic data were recorded, including age, body mass index (BMI), sex, age at onset of disease, smoking status, and current antipsychotic medication. Prior to enrollment patients were examined by an experienced psychiatrist (M.P.) and afterwards underwent an extensive psychiatric and cognitive function assessment using the standardized Greek version of the Positive And Negative Symptom Scale (PANSS) [74] based on the original PANSS [75], the generalized anxiety disorder scale (GAD-7) [76], and the Addenbrooke cognitive test (ACE-R) [77,78].

The PANSS includes 33 items. It is divided into 3 subscales: the positive symptom subscale with seven items (P1–P7), the negative symptom subscale with seven items (N1–N7), and the subscale of general psychopathology with 16 items (G1–G16). The Greek standardized version further includes 3 items (anger intolerance to deferred gratification and emotional volatility; E1–E3) evaluating patients’ perilous behavior. Each item is graded on a 7-point system with higher scores corresponding to incremental levels of psychopathology (1 = absent to 7 = extreme). The final score results from the sum of the positive symptom subscale (7–49), the negative symptom subscale (7–49), and the subscale of general psychopathology (16–112), as well as the subscale for evaluating perilous behavior (3–21) [74].

The GAD-7 consists of 7 anxiety related questions corresponding to symptoms patients might be facing on a day-to-day basis. The questions are based on the diagnostic criteria from the Diagnostic and Statistical Manual of Mental Disorders and graded according to their frequency. Each item is graded from 0 = not at all to 3 = nearly every day [76].

The Addenbrooke Cognitive Examination is a brief 15 min questionnaire exploring five cognitive domains. These include language, verbal fluency, attention, memory, and visuo-spatial ability. The scores on each individual domain are noted and summed up. Higher total score indicates better cognitive function up to a maximum of 100. Scores below 88 are indicative of a diagnosis of dementia [78,79].

The assessment of caffeine dietary intake included questions regarding the varieties of caffeinated products (coffee, tea, chocolate, cocoa, energy drinks), time of consumption throughout the day, quantities consumed per day, and the last time the patients consumed a caffeine-containing product. The dietary intake of caffeine-containing products was assessed by the examiner.

Blood was drawn from all participants for general hematologic and biochemical assays, as well as for measuring the caffeine blood levels and its metabolites, at the time of assessment. The mean time interval of caffeine consumption was 9:00 a.m. The mean time of blood drawing was 12:00 p.m. to control for caffeine consumption (the time of blood drawing varied from late morning to afternoon hours).

To quantify the concentration of caffeine and its metabolites, whole blood samples were centrifuged at 4000 rpm for 5 min, and blood serum aliquots of 1 mL were stored at −20° until they were batch processed. High pressure liquid chromatography with detection under ultraviolet light (HPLC-UV) was employed for the quantification of caffeine and its metabolites. This was a modified version of the method developed by Begas et al. [80]. The lower limit of quantification (LLOQ) for caffeine was 0.125 μg/mL. Participants exhibiting levels of caffeine below the LLOQ were considered non-caffeine consumers.

### 2.5. Variables

Duration of illness was calculated as the time from onset of schizophrenia to participation in the study. Antipsychotic medication was converted to chlorpromazine (CPZ) equivalents [81,82].

### 2.6. Study Size

A sample size of 60 was calculated in the power analysis whilst achieving a confidence level of 99.99% and a ±5% margin of error. This analysis was derived from the global incidence of schizophrenia which is approximately 1% [83,84] and from the population of Larissa which was around 164,095 inhabitants according to the latest survey in 2021 [85].

### 2.7. Statistics

The mean and standard deviation were used to describe continuous variables, whilst numbers and percentages were used to describe categorical variables. Nonparametric statistical tests were deployed. These included the Mann–Whitney U test and the Spearman’s correlation coefficient. All analyses were performed using SPSS version 26 (Chicago, IL, USA).

### 2.8. Ethical Considerations

Study design was in accordance with Declaration of Helsinki. No intervention or experimental treatment were used in the present study. The study protocol was approved by the Ethical Review Board of both the University General hospital of Larissa and the Faculty of Medicine of the University of Thessaly in January 2021.

## 3. Results

In total, 53 patients were included in the present study (Table 1). The mean age of the participants was 45 ± 11 years. The mean age at onset was 23 ± 7 years, while the mean duration of illness from the age of onset was 21.58 years. The majority of patients were males (75%), and 25 patients were current smokers. The mean BMI was calculated at 28.98 ± 6.34 kg/m^2^. Twenty-nine patients were hospitalized due to relapse of schizophrenia, presenting as an acute psychotic episode, and 25 were stabilized for at least 3 months and under regular monitoring on an outpatient basis. The mean CPZ equivalent was measured at 691.83 ± 425.37 mg. The distribution of caffeine serum levels is depicted in Supplementary Figure S1.

### 3.1. PANSS Arm

Table 2 displays the main results regarding the PANSS score. The mean PANSS total score was measured at 72 ± 21. Specifically, patients exhibited 15 ± 8 mean scores in the positive subscore, 21 ± 7 in the negative subscore, 7 ± 4 in the perilous behavior subscore, and 30 ± 9 in the general psychopathology subscore. The median serum caffeine levels were 0.543 ± 2.451 μg/mL.

**Figure 1:**
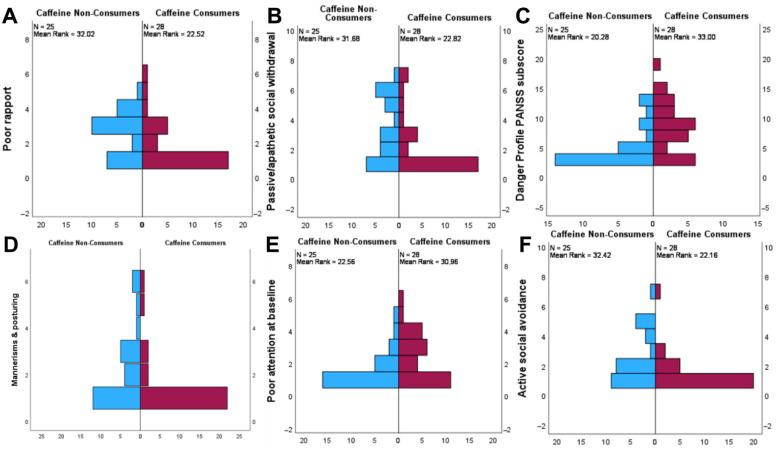
Pyramid plots displaying the difference in distribution amongst caffeine non-consumers and consumers in (**A**) poor rapport, (**B**) passive/apathetic social withdrawal, (**C**) danger profile subscore, (**D**) mannerisms and posturing, (**E**) poor attention, and (**F**) active social withdrawal.

Table 3 presents the statistical results regarding the caffeine consumption, the PANSS subscores, and their subsequent component items. A statistically significant difference in distribution was observed in both the poor rapport (*p* = 0.017) and passive/apathetic social withdrawal (*p* = 0.028) component items of the negative PANSS subscore. Moreover, the perilous behavior PANSS subscore (*p* = 0.002) and its components anger (*p* = 0.013), intolerance to deferred gratification (*p* = 0.026), and emotional volatility (*p* = 0.001) were also found to be statistically significant distributed between caffeine consumers and non-consumers. Finally, mannerism and posturing (*p* = 0.027), poor attention (*p* = 0.033), and active social avoidance showed a statistically significant difference across groups in the general psychopathology PANSS subscore (Figure 1).

In order to further investigate our results, nonparametric correlations using the Spearman’s rho coefficient were carried out (Table 4). A statistically significant positive correlation was found between caffeine levels and the excitement (r = 0.41, *p* = 0.012) and hostility (r = 0.276 *p* = 0.045) components of the positive PANSS subscore. In the same manner, the perilous behavior PANSS subscore (r = 0.435, *p* = 0.001) and its components anger (r = 0.335, *p* = 0.014), intolerance to deferred gratification (r = 0.357, *p* = 0.026), and emotional volatility (r = 0.464, *p* = 0.001) showed a positive correlation with caffeine levels. On the other hand, poor rapport (r = −0.349, *p* = 0.010) and passive/apathetic social withdrawal (r = −0.387, *p* = 0.004) exhibited a statistically significant negative correlation with caffeine levels. In the general psychopathology subscore, poor attention (r = 0.337, *p* = 0.013) exhibited a statistically significant positive correlation with caffeine levels, while active social avoidance (r = −0.347, *p* = 0.011) exhibited a statistically significant negative correlation.

### 3.2. ACE-R Arm

Fifty patients were included in the ACE-R arm (45 ± 11, 13 females and 37 males) (Table 1). The mean Addenbrooke total score was 75 ± 13 (Table 5). Specifically, patients exhibited 16 ± 2 mean scores in the attention and orientation subscore, 16 ± 6 in the memory subscore, 7 ± 3 in the fluency subscore, 22 ± 3 in the language subscore and 13 ± 2 in the visuo-spatial subscore. The median caffeine levels were 0.617 ± 2.503 μg/mL.

**Figure 2:**
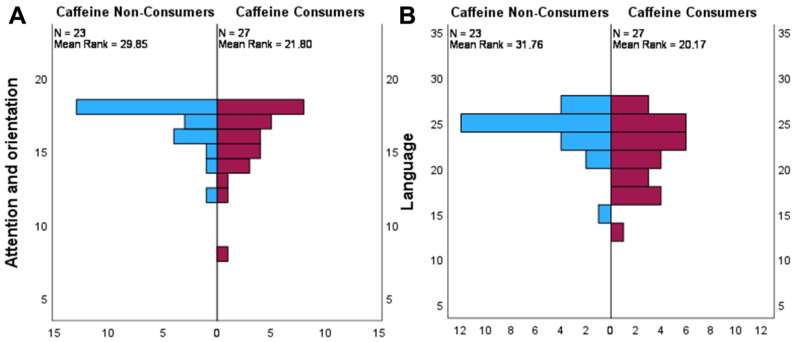
Pyramid plots displaying the difference in distribution amongst caffeine non-consumers and consumers in the (**A**) attention and orientation and (**B**) language ACE-R subscores.

Similarly, a statistically significant difference in distribution of both the attention and orientation (*p* = 0.042) and memory (*p* = 0.005) was found. The ACE-R subscores were found to be statistically significant (Figure 2).

These differences were further explored by nonparametric correlations (Table 6) using the Spearman’s rho correlation coefficient, and a negative correlation was found for both attention and orientation (r = −0.335, *p* = 0.017) and language (r = −0.418, *p* = 0.003) ACE-R subscores and caffeine serum levels.

### 3.3. GAD-7 Arm

A finding worth mentioning is the fact that no correlation was found between the GAD-7 results and caffeine serum levels.

## 4. Discussion

The present observational prospective cohort study investigated a possible correlation between caffeine serum levels, cognitive functions, and symptom severity in patients with schizophrenia in the setting of a psychiatric department in a tertiary hospital. Caffeine serum levels exhibited a positive correlation with the poor attention component of the general psychopathology PANSS subscale, as well as with the attention and orientation component in the ACE-R. Moreover, another positive correlation was observed between the perilous behavior PANSS subscale and caffeine serum levels. Conversely, as caffeine serum levels increased, fewer negative symptoms were reported, specifically, the poor rapport and passive/apathetic social withdrawal of the negative PANSS subscale. In everyday clinical practice, our findings can be translated as patients with more negative symptoms, i.e., anhedonia and apathy, consume more caffeine, conceivably as a form of self-medication while increasing energy and motivation. However, we cannot suggest a clear causative association between aggressive behavior and caffeine consumption, as this correlation can be bidirectional.

Cognitive improvements in healthy individuals in relation to the consumption of caffeine have been reported extensively in the literature. Topyurek et al. [63] note in their invited commentary that cognitive enhancements in healthy individuals vary depending on whether caffeine is administered acutely or chronically. Acute administration of 4 mg/kg body weight [46] or 50/150/250 mg [45] caffeine versus placebo was associated with an increase in sustained attention. On the other hand, in a longitudinal study of 1876 individuals [43] and in a cross-sectional study of 140 individuals [86], chronic consumption of caffeine, measured on a self-report basis, was not associated with any changes in attention.

On the subject of schizophrenia, the only study exploring cognitive functions in patients with schizophrenia and their relationship with caffeine by Nunez et al. [64] associated better cognitive performance in male schizophrenia patients with caffeine consumption regarding domains such as semantic fluency, cognitive speed, working memory, and visual memory. Our results cannot be directly compared with the aforementioned study for several reasons: both genders are included, and different scores assessing cognitive functions were used. However, the fluency and memory ACE-R component exhibited no correlation with caffeine levels even after patients were grouped according to their caffeine consumption habits (caffeine and non-caffeine consumer). Conversely, caffeine serum levels exhibited a negative correlation with the ACE-R attention and orientation component as well as a positive correlation with the poor attention component of the PANSS general psychopathology subscale, i.e., attention worsens, as caffeine serum levels increase which is contrary to caffeine’s effects in healthy populations [45,46], albeit in the acute administration setting. A possible explanation for this phenomenon in this subpopulation could be hypothesized via caffeine’s antagonism on adenosine receptors in the hypofunctioning adenosergic network, which is responsible for arousal, according to the adenosine theory of schizophrenia [34]. Nevertheless, more research is needed to elucidate and to explore this association further.

Aggressive and violent behavior in schizophrenia are terms that exist along the same continuum. Aggression involves verbal threats of harm, while violence involves physical acts [87]. A study by Zislove et al. regarding caffeine consumption and aggression in psychiatric inpatients reported a statistically significant decrease during a one-year period in aggressive behavior, quantified by the need for seclusion of restraint, after the discontinuation of caffeinated beverages sales [88]. A similar study by Carmel [89] observed similar results in a different hospital albeit with no discontinuation of sales policy and highlighted that these differences could possibly arise from methodological differences as well as in discrepancies in reporting mechanisms of aggressive behaviors. It is worth noting that these studies covered all patients hospitalized in psychiatric wards irrelevant of their diagnosis. In the context of patients with schizophrenia, aggression can be quantified according to four standardized Greek PANSS components: hostility, anger, intolerance to deferred gratification, and emotional volatility. Given that the PANSS perilous behavior is used in clinical settings to predict the possible incidence of aggressive behavior in patients with schizophrenia, our results exhibited a positive correlation between all these four aforementioned components and caffeine serum levels, which translates to an increased probability of violent behavior as caffeine serum levels rise. We need to highlight the fact that there is a lack of studies examining the PANSS perilous behavior subscale in the global literature. A possible explanation is the fact that this subscale is only included in the Greek standardized version of the PANSS. Globally, a meta-analysis involving 45,533 patients with schizophrenia concluded that violent behavior in schizophrenia can be determined by elevated positive and total PANSS values [90].

The anxiogenic effects of caffeine are well documented [40,41]; however, no correlation was found between both the anxiety component of the general psychopathology PANSS subscale and the GAD-7 with caffeine serum levels. A cohort study that explored genetic susceptibility mediated via single nucleotide polymorphisms in the adenosine A2A receptor gene (ADORA2A) of 416 healthy individuals towards caffeine’s anxiogenic effects concluded that increasing caffeine consumption diminishes its anxiety-inducing effects irrespective of their genotype [38].

A plethora of studies in the literature report an amelioration of negative symptoms with increasing caffeine consumption. A recent cohort study in France that recruited 804 patients with schizophrenia spectrum disorder reported fewer negative symptoms as per the PANSS with caffeine consumption [73]. It is worth mentioning that coffee consumption was quantified based on patient-reported answers, and this association survived even after controlling for potential confounders such as demographic variables and tobacco consumption. A double-blind placebo study including 13 patients with schizophrenia also showed improvements in negative symptoms after acute caffeine administration. Improvements in withdrawal/retardation, mood, energy, and social involvement were observed after acute administration of 10 mg/kg p.o. caffeine in contrast to placebo (quinine) [69]. Conflicting results, on the other hand, in a large cohort study examining 250 patients with schizophrenia reported no association between caffeine consumption and negative symptoms but with smoking [91]. Patients in the current study exhibited fewer negative symptoms with caffeine serum levels. This association was significant in the poor rapport and passive/apathetic withdrawal component of the PANSS negative subscale and did not change significance when patients were grouped according to their caffeine drinking habits (caffeine consumers/non-consumers) or to their caffeine serum levels. While the connection between negative symptoms of schizophrenia and the adenosine hypothesis are very strong, i.e., patients with schizophrenia should theoretically present with fewer negative symptoms upon amelioration of the hypofunctioning adenosinergic network, no studies to date were able to discover a drug that acts on adenosine receptors with the aim of ameliorating negative symptoms [56].

These results, however, do not imply a causal effect of caffeine on symptom severity or cognition but merely indicate a correlation. Thereby, a different interpretation of these results should be taken into consideration, where caffeine would be consumed as a tool that patients with schizophrenia use to alleviate negative symptoms, or caffeine can be the psychostimulant in a subpopulation with an innate characteristic of aggression. In this context, a qualitative study in Australia reports that caffeine was used by patients with schizophrenia to increase alertness, while patients who reported negative experiences with caffeine decided to self limit its consumption [92]. Therefore, the clinical interpretation of our findings should be individually tailored to each patient and take into account their past history with caffeine consumption.

### Limitations of the Study

Before the generalization of our results, several strengths limitations have to be considered. To the best of our knowledge, our study is one of the first ones investigating the impact of caffeine in schizophrenia patients in Europe and the only one in Greek patients diagnosed with schizophrenia. Furthermore, our results are based on serum level of caffeine measurements that objectively reflect caffeine consumption. On the other hand, the sample size was relatively small, which may limit the generalizability of the findings, making our results preliminary A future direction of our study will include an ROC analysis in order to determine a cut-off point of caffeine serum levels beyond which it is more probable to observe aggressive behavior. Additionally, a significant proportion of participants were smokers, and it is well established that smoking induces cytochrome P450 enzymes such as CYP1A2, which is responsible for the metabolism of caffeine [93]. However, the limited number of non-smokers hinders the ability to perform a multivariate analysis investigating the confounding role of smoking status. Another drawback of our study is based on the fact that our sample included patients with involuntary admission to hospitals and medication non-adherence, which constitute possible factors that drive patients to aggression [94]. Moreover, this was a cross-sectional study with a single assessment per patient, and no follow-up was conducted to monitor potential changes between the clinical picture and caffeine serum levels over time. Future research should consider larger samples with multiple assessment time points to monitor trends in symptomatology and caffeine consumption.

## 5. Conclusions

In summary, the preliminary results of our study highlight the significant associations between caffeine serum levels, symptom severity, and cognition among patients with schizophrenia. While the findings provide valuable insights, they should be interpreted with caution due to the study’s several limitations, the largest being the known effect of smoking on the metabolism of caffeine. More larger scale cohort studies are needed in order to elucidate the impact of caffeine consumption in patients with schizophrenia.

## Figures and Tables

**Table 1:** Demographic characteristics of participants; data are represented as mean ± SD.

Variables		PANSS Arm *n* = 53	ACE-R Arm *n* = 50
Sex	Female	13	13
	Male	40	37
Smoking status	Non-smoker	28	26
	Smoker	25	24
Age (years)		45 ± 11	45 ± 11
Duration of disease (years)		21.58 ± 10.78	21.88 ± 10.80
Body Mass Index (kg/m^2^)		28.98 ± 6.34	29.16 ± 6.37
CPZ equivalent (mg)		691.83 ± 425.37	704.82 ± 426.08
Caffeine levels at baseline (μg/mL)		0.543 ± 2.451	0.617 ± 2.503

**Table 2:** Descriptive patient data in the PANSS arm; data are represented as mean ± SD.

Variable	Measurement
Positive PANSS subscore	15 ± 8
Negative PANSS subscore	21 ± 7
Perilous behavior PANSS subscore	7 ± 4
General Psychopathology PANSS subscore	30 ± 9
Total PANSS score	72 ± 21
Caffeine levels at baseline (μg/mL)	1.589 ± 2.451

**Table 3:** Statistical analysis regarding PANSS variables and caffeine consumption. Mann–Whitney U test results comparing the distribution of symptoms across mean ranks of caffeine non-consumers (*n* = 25) and consumers (*n* = 28). * *p* < 0.05.

Variable	Caffeine Non-Consumers (*n* = 25)	Caffeine Consumers (*n* = 28)	*p*
Positive PANSS subscore	25.1	28.7	0.394
Delusions	27.14	26.88	0.948
Conceptual disorganization	25.56	28.29	0.489
Hallucinatory behavior	24.6	29.14	0.225
Excitement	23.22	30.38	0.055
Grandiosity	26.02	27.88	0.483
Suspiciousness/persecution	27.9	26.2	0.665
Hostility	24.42	29.3	0.128
Negative PANSS subscore	29.74	24.55	0.221
Blunted affect	29.52	24.75	0.248
Emotional withdrawal	27.9	26.2	0.68
Poor rapport	32.02	22.52	0.017 *
Passive/apathetic social withdrawal	31.68	22.82	0.028 *
Difficulty in abstract thinking	24.3	29.41	0.22
Lack of spontaneity and flow of conversation	28.48	25.68	0.492
Stereotyped thinking	26.68	27.29	0.875
Danger Profile PANSS subscore	20.28	33	0.002 *
Anger	22	31.46	0.013 *
Intolerance to deferred gratification	22.26	31.23	0.026 *
Emotional volatility	20.12	33.14	0.001 *
General Psychopathology PANSS subscore	28.66	25.52	0.459
Somatic concern	27.7	26.38	0.66
Anxiety	28.28	25.86	0.543
Guilt feelings	27.98	26.13	0.538
Tension	29.92	24.39	0.139
Mannerisms and posturing	31.26	23.2	0.027 *
Depression	29.6	24.68	0.169
Motor retardation	29.82	24.48	0.129
Uncooperativeness	25.46	28.38	0.349
Unusual thought content	27.18	26.84	0.928
Disorientation	25.24	28.57	0.284
Poor attention	22.56	30.96	0.033 *
Lack of judgement and insight	25.18	28.63	0.409
Disturbance of volition	28	26.11	0.495
Poor impulse control	26.42	27.52	0.738
Preoccupation	24.44	29.29	0.194
Active social avoidance	32.42	22.16	0.008 *
Total PANSS Score	26.56	27.39	0.845

**Table 4:** Spearman’s correlation coefficient of caffeine serum levels across PANSS subscores and its corresponding component items. * *p* < 0.05.

	Spearman’s Rho	*p*		Spearman’s Rho	*p*
Positive PANSS subscore	0.209	0.133	Emotional volatility *	0.464	<0.001
Delusions	0.053	0.706	General Psychopathology PANSS subscore	0.017	0.902
Conceptual disorganization	0.194	0.164	Somatic concern	0.034	0.808
Hallucinatory behavior	0.189	0.176	Anxiety	−0.042	0.766
Excitement *	0.341	0.012	Guilt feelings	−0.1	0.477
Grandiosity	0.161	0.251	Tension	−0.14	0.318
Suspiciousness/persecution	−0.018	0.899	Mannerisms and posturing	−0.227	0.103
Hostility *	0.276	0.045	Depression	−0.145	0.301
Negative PANSS subscore	−0.202	0.147	Motor retardation	−0.207	0.137
Blunted affect	−0.173	0.216	Uncooperativeness	0.178	0.201
Emotional withdrawal	−0.03	0.832	Unusual thought content	0.04	0.777
Poor rapport *	−0.349	0.01	Disorientation	0.178	0.202
Passive/apathetic social withdrawal *	−0.387	0.004	Poor attention *	0.337	0.013
Difficulty in abstract thinking	0.171	0.22	Lack of judgement and insight	0.144	0.302
Lack of spontaneity and flow of conversation	−0.142	0.309	Disturbance of volition	−0.045	0.748
Stereotyped thinking	−0.02	0.887	Poor impulse control	0.147	0.294
Perilous behavior PANSS subscore *	0.435	0.001	Preoccupation	0.238	0.086
Anger *	0.335	0.014	Active social avoidance *	−0.347	0.011
Intolerance to deferred gratification *	0.357	0.009	Total PANSS Score	0.115	0.41

**Table 5:** ACE-R results.

Variables	Measurements
ACE-R attention and memory score	16 ± 2
ACE-R memory score	16 ± 6
ACE-R fluency score	7 ± 3
ACE-R language score	22 ± 3
ACE-R visuo-spatial score	13 ± 2
Total ACE-R score	75 ± 13

**Table 6:** Spearman’s correlation coefficient of caffeine serum levels across ACE-R subscores and its corresponding component items. * *p* < 0.05.

	MW-U Test 23 vs. 27	Spearman’s Correlation Test	
	***p***	**Spearman’s Rho**	***p***
Attention and orientation *	0.042	−0.335	0.017 *
Memory	0.977	−0.002	0.987
Fluency	0.524	0.116	0.423
Language *	0.005	−0.418	0.003
Visuo-spatial perception	0.189	−0.177	0.218
Total ACE-R score	0.501	−0.108	0.455

## Data Availability

The data presented in this study are available on request from the corresponding author due to patient privacy considerations.

## Supplementary Materials

The following supporting information can be downloaded at: https://www.mdpi.com/article/10.3390/brainsci16020209/s1, Figure S1: Distribution curve conveying the frequency distribution of caffeine serum levels in the study sample.
