# Supplementary figures and images for: Association Between Caffeine Levels and Symptom Profile in Schizophrenia: Results from a Cohort Study in Central Greece
Source: Brain Sci. 2026 Feb 10;16(2):209. doi: 10.3390/brainsci16020209 (PMC12939408; doi:10.3390/brainsci16020209)

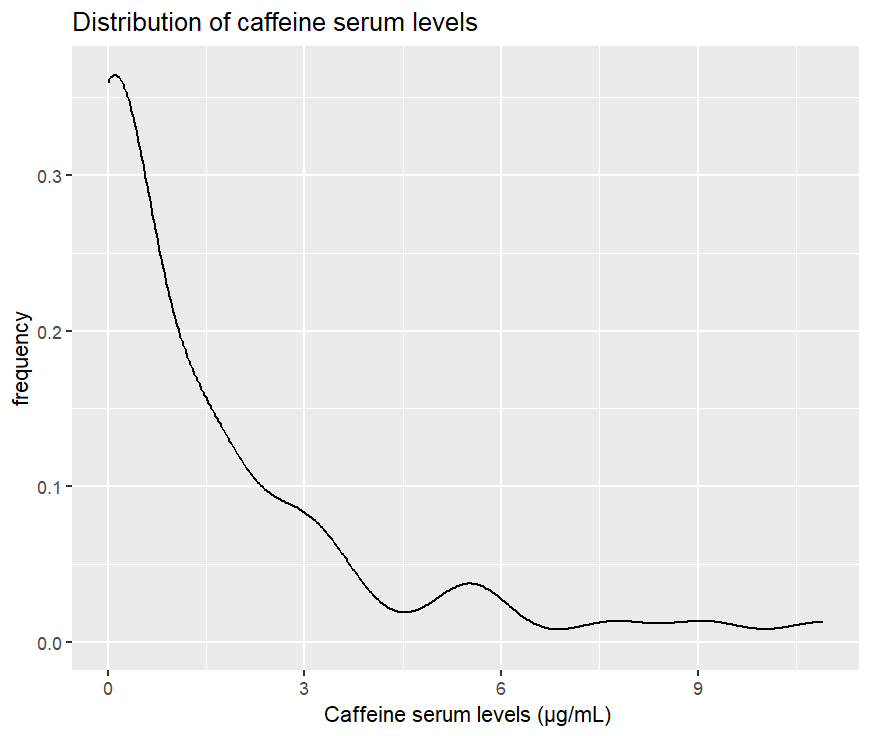

Supplement: Supplementary file 1 [file brainsci-16-00209-s001.zip › brainsci-4117559-supplementary.png]
